# CD4+ T Cell Help Is Mandatory for Naive and Memory Donor-Specific Antibody Responses: Impact of Therapeutic Immunosuppression

**DOI:** 10.3389/fimmu.2018.00275

**Published:** 2018-02-19

**Authors:** Chien-Chia Chen, Alice Koenig, Carole Saison, Suzan Dahdal, Guillaume Rigault, Thomas Barba, Morgan Taillardet, Dimitri Chartoire, Michel Ovize, Emmanuel Morelon, Thierry Defrance, Olivier Thaunat

**Affiliations:** ^1^French National Institute of Health and Medical Research (INSERM) Unit 1111, Lyon, France; ^2^IHU OPERA, Cardioprotection Laboratory, Hospices Civils de Lyon, CIC, Bron, France; ^3^Edouard Herriot University Hospital, Department of Transplantation, Nephrology and Clinical Immunology, Lyon, France; ^4^Lyon-Est Medical Faculty, Claude Bernard University Lyon 1, Lyon, France

**Keywords:** transplantation, transplant immunology, alloimmune response, antibody-mediated rejection, donor-specific antibody, immunosuppression

## Abstract

Antibody-mediated rejection is currently the leading cause of transplant failure. Prevailing dogma predicts that B cells differentiate into anti-donor-specific antibody (DSA)-producing plasma cells only with the help of CD4+ T cells. Yet, previous studies have shown that dependence on helper T cells decreases when high amounts of protein antigen are recruited to the spleen, two conditions potentially met by organ transplantation. This could explain why a significant proportion of transplant recipients develop DSA despite therapeutic immunosuppression. Using murine models, we confirmed that heart transplantation, but not skin grafting, is associated with accumulation of a high quantity of alloantigens in recipients’ spleen. Nevertheless, neither naive nor memory DSA responses could be observed after transplantation of an allogeneic heart into recipients genetically deficient for CD4+ T cells. These findings suggest that DSA generation rather result from insufficient blockade of the helper function of CD4+ T cells by therapeutic immunosuppression. To test this second theory, different subsets of circulating T cells: CD8+, CD4+, and T follicular helper [CD4+CXCDR5+, T follicular helper cells (Tfh)], were analyzed in 9 healthy controls and 22 renal recipients. In line with our hypothesis, we observed that triple maintenance immunosuppression (CNI + MMF + steroids) efficiently blocked activation-induced upregulation of CD25 on CD8+, but not on CD4+ T cells. Although the level of expression of CD40L and ICOS was lower on activated Tfh of immunosuppressed patients, the percentage of CD40L-expressing Tfh was the same than control patients, as was Tfh production of IL21. Induction therapy with antithymocyte globulin (ATG) resulted in prolonged depletion of Tfh and reduction of CD4+ T cells number with depleting monoclonal antibody in murine model resulted in exponential decrease in DSA titers. Furthermore, induction with ATG also had long-term beneficial influence on Tfh function after immune reconstitution. We conclude that CD4+ T cell help is mandatory for naive and memory DSA responses, making Tfh cells attractive targets for improving the prevention of DSA generation and to prolong allograft survival. Waiting for innovative treatments to be translated into the clinical field ATG induction seems to currently offer the best clinical prospect to achieve this goal.

## Introduction

Progress in therapeutic immunosuppression achieved over the last decades has dramatically reduced the incidence of T cell-mediated rejection, which is no longer considered as a significant cause of transplant loss (1).

Unexpectedly, this progress has barely impacted the half-life of transplanted organs that has stagnated over the same period (2). These disappointing results are due to the lack of impact of modern immunosuppressive drugs on the humoral arm of recipient’s alloimmune response (3). Indeed, while under modern immunosuppression regimen, less than 10% of kidney recipients experience an acute cellular rejection episode, the prevalence of *de novo* anti-donor antibodies [donor specific antibody (DSA)] is estimated 10–20% 5 years posttransplantation (4, 5). Consequently, antibody-mediated rejection (AMR) is now widely recognized as the first cause of transplant failure (6–10).

Immunosuppressive drugs used in maintenance therapy mainly act on T cells (3, 11). However, from an immunological point of view, it is surprising that the strict control of cellular (i.e., T cell) immune response obtained with modern immunosuppressive armamentarium did not translate into a more profound impairment of the generation of DSA. Because of their protein nature, HLA molecules are indeed expected to behave as typical T-cell-dependent antigens, which means that donor-HLA specific B cells should be critically dependent upon the help of CD4+ T cells to differentiate into DSA-producing plasma cells (12). This apparent paradox suggests that, in transplantation, some DSA responses might be elicited without the help of CD4+ T cells.

This iconoclastic hypothesis is supported by experimental findings from the group of Zinkernagel that reported the generation of neutralizing antibody against vesicular stomatitis virus in CD4+ T cell-depleted mice (13). Interestingly, dependence on T cell help in this model went decreasing when increasing amounts of protein antigens were recruited to the spleen, leading the authors to conclude that both antigen dose and localization in secondary lymphoid organs are key to circumvent T cell help for induction of B cell responses (13).

It is noteworthy that organ transplantation could meet these two conditions since donor specific HLA molecules are highly expressed by the endothelial cells of graft vasculature, which is directly connected to recipient’s vessels.

We, therefore, undertook this study to test whether transplant recipients could generate DSA in the absence of CD4+ T cell help.

## Materials and Methods

### Human Study

To determine the capacity of T cells to get activated under immunosuppression, we prospectively enrolled 22 patients who underwent kidney transplantation at the Lyon University Hospital between 2015 and 2016. Inclusion criteria were (i) first transplantation (kidney or kidney–pancreas), (ii) no anti-HLA antibody at the time of the transplantation. These 22 patients were compared to 9 healthy controls.

Whole blood samples were collected by venepuncture into heparin-containing vials, once in controls and before transplantation and at 3 months and 1 year after transplantation for transplanted patients.

Peripheral blood mononuclear cells (PBMCs) and plasma were isolated by Ficoll gradient centrifugation. Plasma was then centrifuged at 4,000 g for 10 min to remove platelets. PBMCs were plated 1 h in petri dishes to discard adherent cells (monocytes) and then 1 × 10^6^ non-adherent cells were cultured 24 h at 37°C in 5% CO_2_ in 1 mL of patient’s own plasma (containing or not immunosuppressive drugs) in the presence or absence of human T-activator CD3/CD28 beads (Gibco Dynabeads^®^). For IL21 staining, 1 µL of Brefeldin A (BD Bioscience) was added for the last 5 h of the culture. After 24 h of culture, Dynabeads were removed with a magnet and PBMCs were analyzed by flow cytometry as detailed below.

This study was carried out in accordance with French legislation on biomedical research. All subjects gave written informed consent in accordance with the Declaration of Helsinki. The protocol and the biocollection were authorized by the Ministry of Research and the Rhône-Alpes Regional Health Agency (#AC-2011-1375 and #AC-2016-2706).

### Mice

Wild-type C57BL/6 (H-2^b^) and BALB/c (H-2^d^) mice were purchased from Charles River Laboratories (Saint Germain sur l’Arbresle, France).

C57BL/6-Tg(CAG-EGFP)1Osb/j (GFP) mice were purchased from The Jackson Laboratory (Bar Harbor, ME, USA).

MHC II knock out (AßKO) mice on C57BL/6 genetic background were provided by Dr. Benoist and Mathis (Boston, MA, USA) (14).

HLA A2 transgenic mice on C57BL/6 genetic background (15) were kindly provided by Dr Lemonnier (Paris, France).

RAG2 Knock Out (RAG2 KO) mice on C57BL/6 background came from CDTA (Cryopreservation Distribution Typage et Archivage animal, Orléans, France). Mice 8–15 weeks of age were used and maintained under specific pathogen-free conditions in our animal facility: Plateau de Biologie Expérimentale de la Souris (PBES, ENS Lyon, France).

This study was carried out in accordance with the French legislation on live animal experimentation. The protocol was approved both by local “CECCAPP” (http://www.sfr-biosciences.fr/ethique/experimentation-animale/ceccapp) and national AFiS ethical committees (#02870.01).

### Models of Allosensitization

#### Skin Grafting

Skin grafting was performed using the method described by Billingham et al. (16). Briefly, a piece of skin graft about 1 cm × 1 cm in size was harvested from the trunk of donor and it was then implanted on the back of the recipient, fixed by silk sutures, and protected for 7 days with bandage.

#### Heterotopic Heart Transplantation

Cervical heterotopic heart transplantations were performed as in Chen (17). Briefly, cardiac allografts were transplanted into subcutaneous space of right neck. Anastomoses were performed by connecting end-to-end the ascending aorta of the graft with the recipient’s common carotid artery and by pulling the main pulmonary artery with the external jugular vein.

#### Intravenous Allogeneic Cell Injection

To mimic the allosensitization that results from solid organ transplantation, 10 × 10^6^ splenocytes from HLA A2 mice were injected intravenously to wild-type C57BL/6. In contrast with subcutaneous injection, IV injection of allogeneic splenocytes indeed allows delivering a high quantity of alloantigen to the spleen of recipient (Figure S1A in Supplementary Material) and triggered DSA generation (Figure S1B in Supplementary Material).

### CD4+ T Cell Depletion *In Vivo*

Seminal experimental studies addressing the role of helper T cells in rejection *in vivo* have relied on the treatment of wild-type (WT) mice with depleting anti-CD4 monoclonal antibodies (mAbs) (18, 19).

In the present study, we used IP administration of GK1.5, a rat IgG2b anti-murine CD4 mAb (20), commonly used to induce CD4+ T cell depletion in mice (21).

### Cell Lines

The human erythroleukemia cell line K562 (22) lacking expression of all MHC I and II molecules and the single antigen expressing cell Line (SAL) A2, a K562 cell line transfected with plasmid coding for HLA A2 and selection genes for the resistance to neomycin and ampicillin (23) were kindly provided by Dr. Doxiadis (Leiden, The Netherlands).

K562 cells were cultured in RPMI-1640 (Invitrogen) complemented with fetal calf serum 10% (Dutscher), l-Glutamine 2 mM (Invitrogen), penicillin 100 U/mL, streptomycin 100 µM, and HEPES 25 mM (Invitrogen).

Single antigen expressing cell line A2 cells were cultured in the same medium as K562 complemented with G418, a neomycin derivative (Invitrogen) at the concentration of 1.75 mg/mL.

### Flow Cytometry

After a 24 h culture, human PBMCs were stained 30 min at room temperature in the dark with the relevant fluorescent antibodies directed against CD3 (UCHT1), CD4 (SK3), CD8 (SK1), CD25 (2 A3), CXCR5 (RF8B2), CD40L (TRAP1), IL21 (3A3-N2.1), ICOS (ISA-3); all from BD Biosciences except ISA-3 from ebioscience. Cells were then stained with and a viability dye LIVE/DEAD Aqua (Invitrogen) according to the manufacturer’s instructions and fixed with Cytofix/Cytoperm^®^ fixation/permeabilization kit (BD Biosciences) before analysis.

A FACS ARIA II flow cytometer was used for flow cytometry. Data were analysed with BD FACS Diva software (BD Biosciences).

Murine single-cell suspensions from spleen, lymph nodes, thymus, or blood were incubated with a blocking anti-mouse Fc receptor antibody (2.4G2, home-made hybrydoma). Cells were then incubated at 4°C with relevant fluorescent antibodies: CD3 (145-2C11), CD4 (RM4-4), CD8 (53-6.7) and CD19 (1D3) (all from BD Biosciences). Before analysis by flow cytometry, DAPI (4′,6-diamidino-2-phenylindole dihydrochloride, Sigma-Aldrich) was added to the cell suspension to exclude dead cells. Samples acquisitions were made on a BD LSR II flow cytometer (BD Biosciences). Data were analyzed with FlowJo software (Tree Star).

### Cell Sorting and Adoptive Transfer of B Lymphocytes

For isolation of B lymphocytes, splenocytes from immunized mice were stained with phycoerythrin (PE)-conjugated antibodies against CD3ε (145-2C11), CD4 (RM4-5), CD8 (53-6.7), CD11b (M1/70), CD11c (HL3), NK-1.1 (PK136), Ter119/Erythroid cells (TER-119), CD117 (2B8), and PerCP-Cy5.5 conjugated antibody against B220 (RA3-6B2) (all from BD Biosciences). After staining, cells were negatively separated by LD magnetic columns with anti-PE Microbeads labeling (Miltenyi Biotec). The separated cell suspensions underwent cell sorting by using a BD FACS Aria cell sorter (BD Biosciences) to collect PE negative and PerCP-Cy5.5 positive cells. 5 × 10^6^ purified B lymphocytes were transferred to RAG2 KO mice by intravenous injection.

### Detection of Anti-HLA A2 Antibodies

1 × 10^5^ SAL A2 or K562 (negative control) cells were incubated with mice sera diluted at 1/1,000 for 30 min at 4°C. After washing, cells were stained with PE-conjugated anti-light chain antibody (187.1) (BD Biosciences) and analyzed by flow cytometry. To rule out the possibility to miss a low titer of anti-HLA A2 antibodies, all negative sera were systematically screened again at a 1/100 dilution. The titer of anti-HLA A2 antibodies at each time point (dx) was calculated with the following formula:

$$\mathrm{normalised DSA titer}=\frac{{\mathrm{MFI}}_{\mathrm{SAL}}\left(dx\right)/{\mathrm{MFI}}_{K562}\left(dx\right)}{{\mathrm{MFI}}_{\mathrm{SAL}}\left(d0\right)/{\mathrm{MFI}}_{K562}\left(d0\right)}$$

The normalized DSA titer, therefore, reflects the fold increases of specific signal over the baseline (measured before transplantation).

### Detection of Anti-HLA A2 Specific B Cells by ELISpot Assay

Single-cell suspensions were stained with PE-conjugated anti-B220 (RA3-6B2) (BD Biosciences). B cells were positively separated by passing through LS magnetic column with anti-PE microbeads labeling. After separation, cells underwent *in vitro* activation with a mixture of the TLR7/8 ligand R848 at 1 µg/mL and recombinant mouse IL-2 at 10 ng/mL (Mabtech) for 72 h. Then, cells were cultured for 24 h in the 96-well plates precoated with capture anti-IgG antibody (ELISpot^PLUS^ mouse IgG kit, Mabtech). Detection of spots was performed by adding either anti-IgG biotinylated detection antibody (Mabtech) or biotin-labeled HLA-A*02:01 Pentamer (ProImmune) for 2 h, followed by incubation with streptavidin-ALP (Mabtech) for 1 h and development by BCIP/NBT-plus substrate (Mabtech).

### Detection of Anti-NP Antibodies by ELISA

For some experiments, mice were immunized intraperitoneally with 75 µg NP-KLH mixed with 100 µL Inject Alum Adjuvant (Thermo Scientific, Courtaboeuf, France) or 200 µg NP-Dextran. Preimmune sera and sera obtained once a week after immunization were tested for IgM and IgG anti-NP antibodies. Maxisorp plates (Nunc) were coated with NP 23-conjugated BSA. Serially diluted serum samples were added for 1 h 30 at room temperature. Anti-NP IgM and IgG Abs were detected using alkaline phosphatase conjugated goat anti-mouse IgM or IgG Abs (1/2,000 dilution) followed by phosphatase substrate (Sigma-Aldrich). The plates were then read at 405 nm/490 nm with an automatic reader (Zeiss VERSAmax). OD was converted to concentration based on standard curves with sera from C57BL/6 mice immunized with NP-KLH using a four-parameter logistic equation (Softmax Pro 5.3 software; Molecular Devices).

### Statistical Analyses

Statistical significance of differences was tested with chi-square test for proportions and non-parametric tests for continuous variables: Mann–Whitney or Kruskal–Wallis with Dunn’s multiple comparisons for unpaired dataset and Friedman with Dunn’s multiple comparisons for paired dataset.

Skin graft survivals were compared using the log-rank test.

The differences between the groups were considered statistically significant for *p* < 0.05 and were reported as asterisk symbols (*p < 0.05; **p < 0.01; ***p < 0.001; ****p < 0.0001).

## Results

### Presentation of the Experimental Model

**Figure 1:**
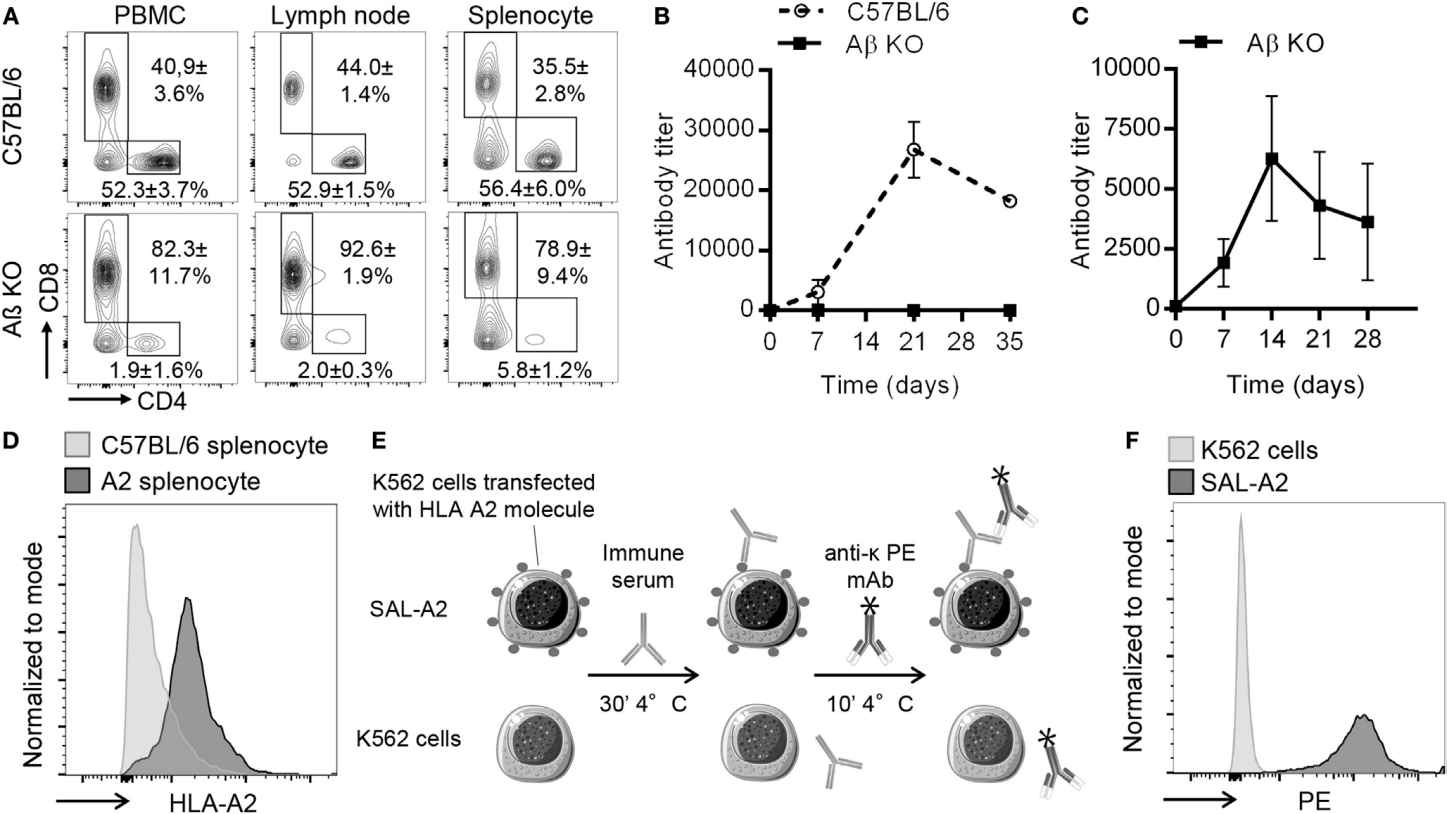
Presentation of experimental model. **(A)** Representative flow cytometry profiles of CD3+ T cells in PBMC and secondary lymphoid organs is shown for wild-type C57BL/6 (upper row) and AβKO (lower row) mice. Mean and SD from analysis of 10 animals of each strain are indicated for the % of CD4+ and CD8+ T cells. **(B)** Evolution over time of anti-NP IgG titer (mean ± SD) after immunization with the thymo-dependent model antigen NP-KLH is shown for wild-type C57BL/6 (*n* = 3, dashed line) and AβKO (*n* = 3, black line). **(C)** Anti-NP antibody titer was measured by ELISA in the circulation of AβKO mice (*n* = 3) before and every 7 days postimmunization with the thymo-independent model antigen NP-dextran (mean ± SD). **(D)** Splenocytes from C57BL/6 (negative controls) and HLA A2 transgenic mice were incubated with fluorescent-conjugated mice anti-human HLA I monoclonal antibody (mAb) (W6/32). Representative flow cytometry histograms are shown. **(E)** To quantify donor specific antibody (DSA), diluted sera were incubated with K562 or single antigen expressing cell line (SAL)-A2 for 30 min at 4°C. Cells were washed and incubated with a phycoerythrin (PE)-conjugated anti-light chain mAb before measurement of PE mean fluorescence intensity by flow cytometry. Anti-A2 antibody titer was normalized as explained in Section “Materials and Methods.” The normalized DSA titer reflects the fold increases of specific signal over the baseline. **(F)** Representative examples of cytometry profiles obtained after incubation of K562 and SAL-A2 with an anti-A2 immune serum are shown.

AβKO mice, which are genetically modified C57BL/6 mice devoid of CD4+ T cells were used as recipients. AβKO mice lack major histocompatibility complex class II (MHCII) molecules (14). As a result, positive selection of CD4+ T cells can not occur in the thymus and AβKO mice show near-complete elimination of CD4+ T lymphocytes in the periphery, the lymph nodes, and the spleen (Figure 1A). The few remaining detectable CD4+ T cells are anyhow unable to provide efficient help to B cells because the latter do not express MHCII. This was demonstrated by the complete abrogation of the IgG response to a NP-KLH, a model of thymo-dependent antigen (24) (Figure 1B). Importantly, this lack of IgG response in AβKO mice was not due to an abnormal B cell compartment since (i) B-lymphocytes occur in normal numbers and are capable of terminal differentiation to plasma cells in these animals (14), and (ii) we showed that AβKO mice indeed generated NP-specific IgM in response to vaccination with NP-dextran (Figure 1C), a model of thymo-independent antigen (25).

To simplify the monitoring of donor-specific antibody (DSA) response in recipient mice we used donors with the same genetic background (C57BL/6, H2^b^) except for the transgenic expression of the human MHC I molecule HLA-A2 [kind gift from Dr Lemonnier (15); Figure 1D].

In this model, the DSA response, which is exclusively directed against the HLA-A2 molecule was easily monitored using single antigen-expressing (SAL-A2) cell line [kind gift from Doxiadis (23)] and the sensitive and specific flow cross match assay (Figures 1E,F), a technique routinely used for the follow-up of transplant recipients in the clinic (26).

### CD4+ T Cell Help Is Mandatory for Naive DSA Response

**Figure 2:**
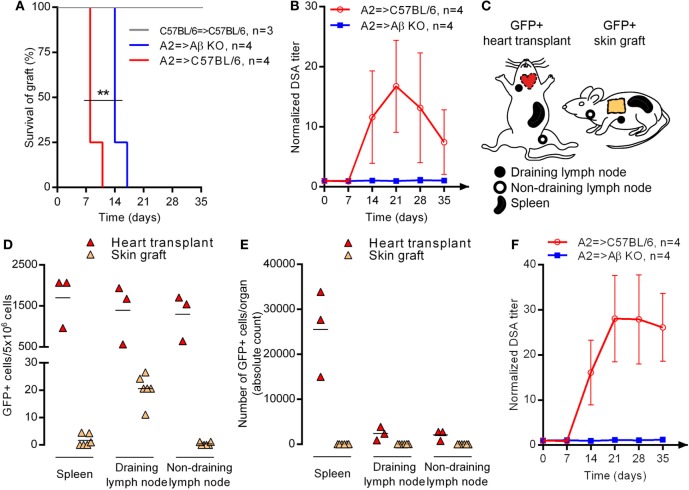
Site of allosensitization and CD4+ T cell help for naive donor-specific antibody response. **(A)** Skin from C57BL/6 or HLA-A2 transgenic (A2) donor was grafted to C57BL/6 or AβKO recipient. Survivals of skin graft were compared between the three groups using Log rank test. **(B)** A2 skin was grafted to C57BL/6 or AβKO recipients. Evolution of normalized donor specific antibody (DSA) titer (mean ± SD) in the circulation of recipients is shown. Log-rank test; **p < 0.01. Skin graft or heart transplant from GFP+ transgenic donor was grafted/transplanted to C57BL/6 recipient. **(C)** The location of draining and non-draining lymph node according to the sensitization procedure is shown on the scheme. Two days post-procedure the percentage **(D)** and absolute number **(E)** of GFP+ cells was enumerated by flow cytometry in the secondary lymphoid organs of recipients (each symbol represents a mice, mean is indicated). **(F)** A2 heart was transplanted to C57BL/6 or AβKO recipients. Evolution of normalized DSA titer (mean ± SD) in the circulation of recipients is shown.

Because AβKO mice have a normal CD8+ T cell compartment (Figure 1A), they efficiently rejected an allogeneic A2 skin graft with only a slight delay as compared with C57BL/6 WT controls (respective mean survival 14.75 ± 1.5 vs 8.75 ± 1.5 days, Log Rank: *p* = 0.0058; Figure 2A).

Donor-specific antibody response was monitored in naive recipients grafted with an A2 skin. Anti HLA-A2 alloantibodies appeared in the serum of WT recipients between day 7 and 14 posttransplantation, peaked at day 21, and decreased slowly thereafter with the elimination of alloantigens due to the rejection process (Figure 2B). No DSA could be detected in the serum of AβKO.

These results confirm the conclusion of Steele et al. (27) who reported that CD4+ T cells are essential helper cells for B cell alloantibody production in a similar murine model of MHC-disparate skin graft rejection. However, in the study by Ochsenbein et al., dependence on T cell help went decreasing when increasing amounts of antigen were recruited to the spleen (13). One possibility to explain the lack of DSA response in AβKO recipients grafted with A2 skin could be that this procedure provides too few alloantigens drained to the spleen of recipients (in contrast with transplantation, no vascular anastomosis is performed between the circulations of donor and recipient during grafting). To test this hypothesis, C57BL/6 donor mice transgenic for the green fluorescent protein (GFP+) were used as donor of skin graft or heart transplant (Figure 2C). The proportion (Figure 2D) and absolute number (Figure 2E) of GFP positive cells were quantified by flow cytometry at day 2 post-procedure in the draining lymph node, a distant non-draining lymph node, and the spleen of recipients. After skin grafting, only few GFP+ cells were detected, exclusively in the draining lymph node of recipient (Figures 2D,E). About 100 times more GFP+ cells were found in the draining lymph node of heart recipients. In addition, GFP+ cells were also detected in the spleen and the non-draining lymph node after heart transplantation (Figure 2D), demonstrating that alloantigens are drained trough recipient’s circulation after transplantation but not after grafting. In fact, when absolute numbers were considered, spleen appeared as the main site of allosensitization after transplantation (Figure 2E).

Despite these differences in alloantigen dose and localization in secondary lymphoid organs of recipients, we could not detect any DSA generation after A2 heart transplantation in AβKO recipients (Figure 2F). We conclude from this set of experiments that DSA generation in naive recipients requires CD4+ T cell help.

### CD4+ T Cell Help Is Mandatory for Memory DSA Response

In contrast with laboratory mice, the immune system of which is largely naïve (28), transplanted patients have an immune memory, including memory B cells (29). Interestingly, some studies have reported that ability of virus-specific memory B cells to secrete IgG is independent of cognate or bystander T cell help (30).

**Figure 3:**
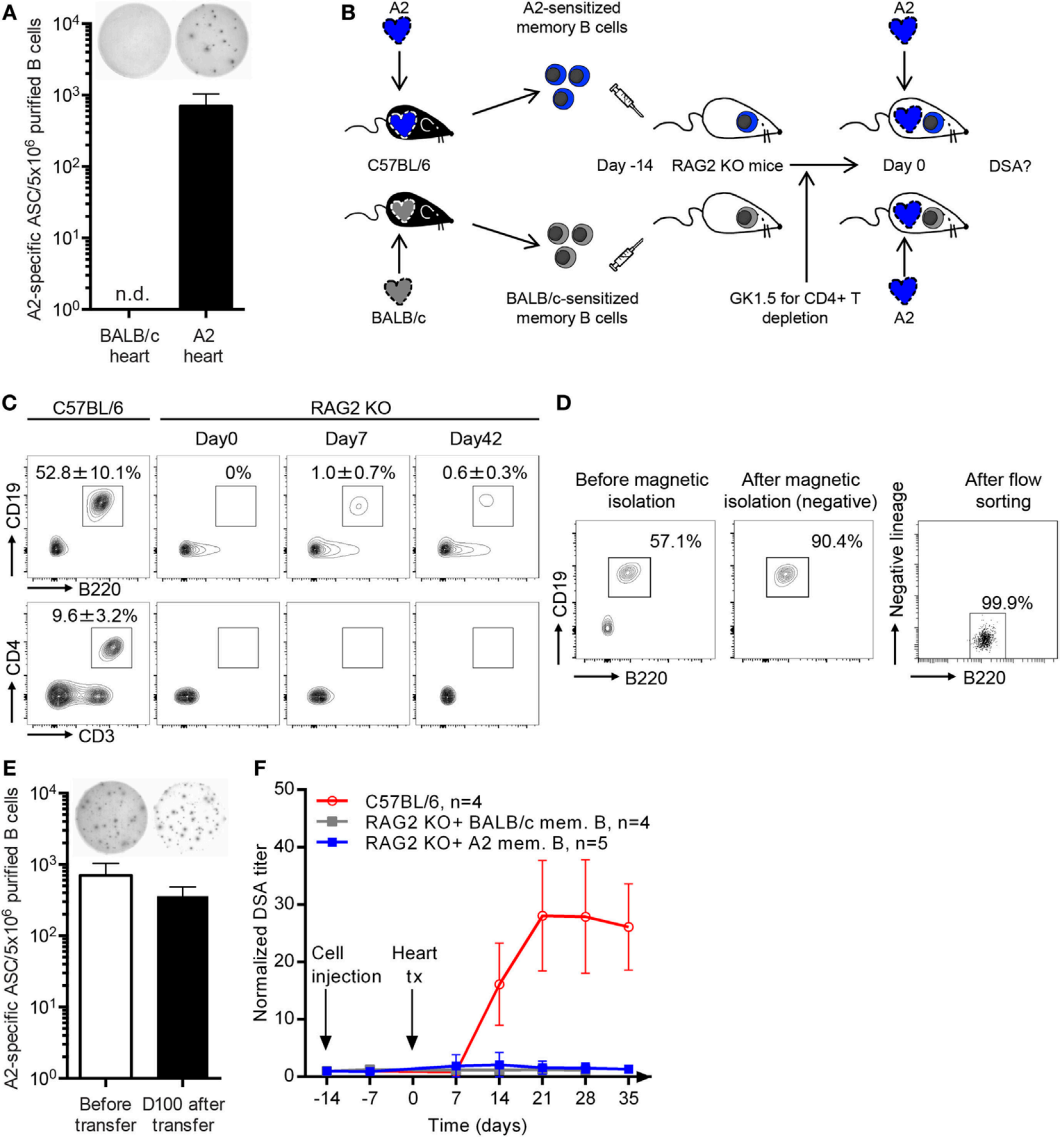
CD4+ T cell help is mandatory for memory donor-specific antibody (DSA) response. **(A)** Allogeneic heart from HLA A2 transgenic (A2) or BALB/c donor was transplanted to C57BL/6 recipient. Fifty days posttransplantation HLA A2-specific memory B cells were enumerated in the spleen of recipients by ELISpot. Representative wells are shown and the number of A2-specific memory B cells (mean ± SD) is plotted for the two groups (*n* = 4). **(B)** Graphical representation of the experimental setting used to evaluate the importance of CD4+ T cell help in memory DSA response. **(C)** Flow cytometry was used to quantify the proportion (mean ± SD) of B cells (B220+CD19+) and helper T cells (CD3+CD4+) in controls (C57BL/6; left column) and RAG2 KO recipients before memory B cell transfer (day 0, second column from the left), just prior heart transplantation (day 7, third column from the left), and 42 days after A2 heart transplantation (right column). **(D)** The purity of B cell suspension, obtained from the spleen of C57BL/6 mice sensitized with a A2 or a BALB/c heart transplantation, was evaluated by flow cytometry: before magnetic isolation (left panel), after magnetic isolation (middle panel) and after flow sorting (just prior transfer to RAG2KO mice, left panel). **(E)** HLA A2-specific memory B cells were enumerated by ELISpot in B cell suspensions before transfer (left histogram) and in the spleen of RAG2 KO animals transplanted with an A2 heart 100 days after transfer (right histogram). **(F)** Evolution of normalized donor-specific antibody titer (mean ± SD) was monitored in the circulation of 3 groups of recipients transplanted with an A2 heart: wild-type C57BL/6 (positive controls, dotted line), RAG2 KO transferred with anti-BALB/c memory B cells (negative controls, dashed line), and RAG2 KO transferred with anti-A2 memory B cells (experimental group, black line).

Transplantation with an A2 but not a BALB/c heart leads to the generation of anti-A2 specific memory B cells that could be readily quantified in recipients’ spleen with ELISpot assay (Figure 3A). To determine whether the rechallenge of allospecific memory B cells can lead to the generation of DSA in the absence of CD4+ T cells, we passively transferred A2- or BALB/c-specific memory B cells to C57BL/6 RAG2 KO recipients (Figure 3B). C57BL/6 RAG2 KO mice lack the recombinase necessary for TCR and BCR rearrangements and are, therefore, devoid of T and B cells (Figure 3C). In addition to the high degree of purity achieved when isolating B cells (99.83 ± 0.21%, Figure 3D), we ensured that no CD4+ T cell remained in C57BL/6 RAG2 KO post-cell transfer by administering anti-CD4 mAb (Figure 3B). As expected, B cells (but neither CD4+ nor CD8+ T cells) could be detected in the circulation of transferred C57BL/6 RAG2 KO animals up to the end of the follow-up period (Figure 3C). Not only were B cells detectable in transferred C57BL/6 RAG2 KO mice, but A2-specific memory B cells were able to differentiate into DSA-producing plasma cells, as demonstrated by ELISpot assays conducted with splenocytes harvested 100 days post-transfer (Figure 3E).

However, and in striking contradiction with our hypothesis, the rechallenge of allospecific memory B cells with A2 heart did not lead to the generation of DSA in the absence of CD4+ T cells (Figure 3F). The mandatory nature of CD4+ T cell help for DSA generation in sensitized recipients was further confirmed by demonstrating that co-transfer of purified CD4+ T cells with memory B cells in C57BL/6 RAG2 KO mice was sufficient to restore alloantibody response after rechallenge (Figures S1C,D in Supplementary Material).

### Impact of Maintenance Immunosuppression on T Follicular Helper Functions in the Clinic

Based on our previous results, it seems clear that DSA generation, both in naive and sensitized recipients, requires help from CD4+ T cells. The fact that, despite therapeutic immunosuppression, 10–20% of transplant recipients develop *de novo* DSA within 5 years posttransplantation (3–5), suggests that immunosuppressive drugs do not efficiently block CD4+ T cell help to B cells.

**Figure 4:**
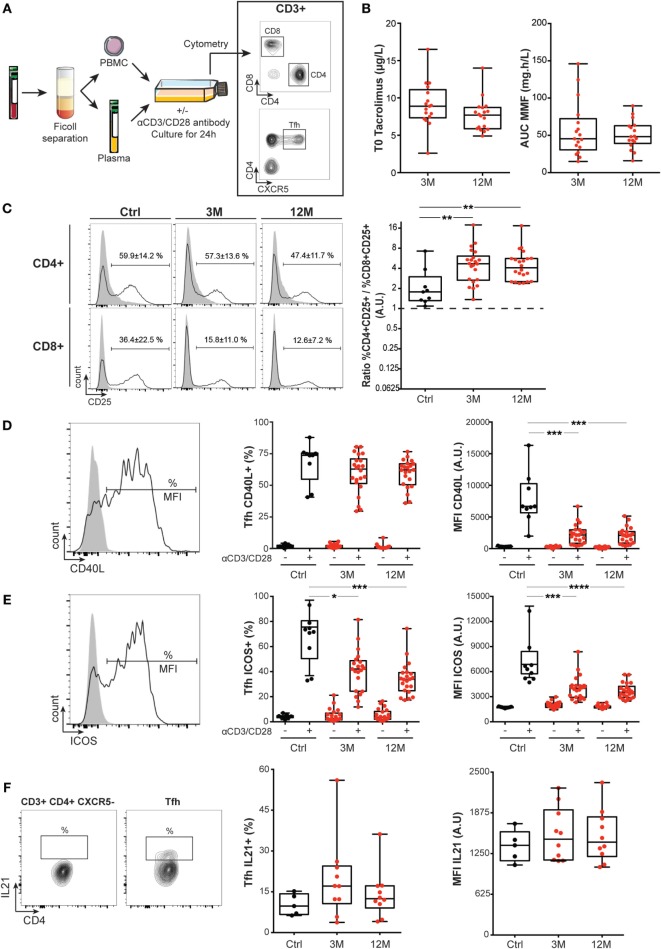
Impact of maintenance immunosuppression on CD4+ T cell activation in the clinic. **(A)** T cells were prospectively monitored in the circulation of 9 healthy controls and 22 patients after renal transplantation (3 months, 3M; and 12 months, 12M). Peripheral blood mononuclear cell (PBMC) and plasma (containing or not immunosuppressive drugs) were separated by Ficoll. **(B)** Tacrolimus trough levels (T0) and areas under the curve (AUC) of mycophenolate mofetil measured in the plasma of renal transplanted patients at 3 and 12M are plotted (each symbol is a patient). **(C)** Left panel: representative FACS profile of controls (Ctrl; left column) and renal recipients (3 M, middle column; 12 M, left column) The expression of the activation marker CD25 was measured by flow cytometry on the surface of CD4+ (top) and CD8+ (bottom) T cells. The analysis was performed after 24H culture in patient’s own plasma without (gray area) or with (black line) stimulation with CD3/CD28 microbeads. The percentage of CD25-expressing T cells after stimulation is indicated (mean ± SD). Right panel: the ratio of the percentage of CD4+ T cells expressing CD25 over the percentage of CD8+ T cells expressing CD25 after stimulation is plotted for the 9 controls (Ctrl, black symbols) and the 22 renal recipients at 3 and 12M (red symbols). Ratios were compared between Ctrl and renal recipients at M3 and M12: Kruskal–Wallis with Dunn’s multiple comparisons. **p < 0.01. **(D,E)** Flow cytometry was used to analyze the expression of helper molecules CD40L **(D)** or ICOS **(E)** on T follicular helper cells (Tfh) surface after 24H culture in control’s patient’s own plasma without (gray area) or with (black line) stimulation with CD3/CD28 microbeads. Left panel: representative FACS profile. Middle panel: percentage of Tfh that express the helper molecule. Right panel: level of expression of the helper molecule [mean fluorescence intensity (MFI)]. Each control (Ctrl, black) and renal recipient at 3 and 12M (red) is a symbol. **(F)** Flow cytometry was used to analyze the production of IL21 by Tfh in controls (Ctrl, black symbols) and renal recipients at 3 and 12M posttransplantation (red symbols) for whom frozen PBMCs were available. Left panel: representative FACS profile. Middle: percentage of IL21-expressing Tfh. Right: level of expression of IL21 (MFI). Percentage of positive Tfh and MFI were compared between Ctrl and renal recipients at M3 and M12: Kruskal–Wallis with Dunn’s multiple comparisons. *p < 0.05; ***p < 0.001; ****p < 0.0001.

To test this hypothesis, we prospectively monitored circulating T cells in 22 immunosuppressed renal recipients, 3 months (3 M) and 12 months (12 M) posttransplantation (Figure 4A). Clinical characteristics of the cohort are presented Table 1 and the trough levels of maintenance immunosuppressive drugs are shown Figure 4B. Immunosuppressed patients were compared to 9 age- and sex-matched healthy controls. Indeed, comparing the evolution of T cell parameters before *versus* after transplantation for the same patients would not have allowed to disentangle the impact of immunosuppressive drugs from that of the correction of end stage renal failure, which has a major impact on the immune system (31).

**Table 1:** Characteristics of study population.

Recipients*n*(%) or mean ± SD	All pts	ATG	BasiliX	*p*-Value*

	22 (100)	11 (50)	11 (50)	
Age at transplantation (years)	47 ± 13	47 ± 16	47 ± 9	0.99
Men	14 (64)	6 (55)	8 (73)	0.66
Cause of ESRD				0.09
Glomerulonephritis	7 (31)	3 (27)	4 (36)	
Diabetes mellitus	8 (36)	6 (55)	2 (18)	
ADPKD	3 (14)	2 (18)	1 (9)	
Other	4 (18)	0 (0)	4 (36)	
Duration of dialysis before Tx (months)	22 ± 14	23 ± 12	21 ± 16	0.38
**Donors**				
Donor age (years)	47 (13)	43 ± 14	47 ± 9	0.43
Men	13 (50)	6 (55)	4 (36)	0.67
Deceased donor	21 (81)	10 (91)	8 (73)	0.47
Expanded criteria donor	11 (42)	5 (45)	4 (36)	1
**Transplantation**				
Number of HLA MisMX (A, B, DR)	3.7 ± 1.6	4.3 ± 1.5	3.2 ± 1.6	0.10
Cold ischemia time (hours)	11 ± 6	12 ± 4	11 ± 6	0.47
Induction therapy				Not tested
Antithymocyte globulin (rabbit)	11 (50)	11 (100)	0 (0)	
Basiliximab	11 (50)	0 (0)	11 (100)	
Maintenance immunosuppression				0.54
Tacrolimus	16 (73)	9 (82)	7 (66)	
Cyclosporin	6 (27)	2 (18)	4 (36)	
Everolimus	2 (9)	0 (0)	2 (18)	
Mycophenolate Mofetil	20 (91)	11 (100)	9 (82)	
Corticosteroids	22 (100)	11 (100)	11 (100)	

*ADPKD, autosomal dominant polycystic kidney disease; ATG, antithymocyte globulin; BasiliX, basiliximab; ESRD, end-stage renal disease; MisMX, mismatches*.

*Comparison between patients induced with ATG and basiliximab: chi-square test for comparison of proportions and Mann–Whitney test for comparison of continuous variables.

To test whether maintenance immunosuppressive regimen is able to block T cells activation, PBMCs of patients were cultured with anti-CD3/CD28 microbeads in the presence of patient’s own plasma (Figure 4A). Surface expression of the α chain of IL-2 receptor (CD25) was used to monitor activation of CD8+ and CD4+ T cells (32). As expected, *in vitro* stimulation of T cells from healthy controls led to significant upregulation of CD25 expression on both CD4+ and CD8+ subsets (Figure 4C). Interestingly, while the presence of plasma containing immunosuppressive drugs significantly reduced activation-induced upregulation of CD25 on CD8+ T cells, it had no significant impact on CD4+ T cells (Figure 4C).

To further document the lack of impact of maintenance immunosuppression on thymo-dependent humoral immune response, we focused our analysis on T follicular helper cells (Tfh), a CD4+ T cell subset specialized for providing help to B cells (33, 34). Recent studies have indeed demonstrated that CD4+CXCR5+ human blood T cells represent the circulating compartment of Tfh (35), and we confirmed that CD3+CD4+CXCR5+Tfh could be detected in the circulation of both controls and transplanted patients (Figure 4A).

T follicular helper cells provide help to B cells through a variety of molecules that are either expressed on their surface: CD40L (36) and ICOS (37), or are secreted: IL-21, a type I cytokine recognized as the most potent driver of B cell terminal differentiation (38, 39). *In vitro* activation with anti-CD3/CD28 microbeads promoted the expression of CD40L (Figure 4D) and ICOS (Figure 4E) by the Tfh. As expected, triple maintenance immunosuppression significantly decreased the level of expression of both CD40L and ICOS on activated Tfh (Figures 4D,E). However, the impact of triple maintenance immunosuppression was incomplete because the drug combination failed to reduce the percentage of Tfh that expressed CD40L after stimulation (Figure 4D) or the production of IL21 by Tfh (Figure 4F).

### Reduction of CD4+ T Cells Number Dampens Naive and Memory DSA Responses

Transplanted patients often receive induction therapy at the time of transplantation to prevent acute rejection during the early posttransplantation period. Induction immunosuppressive agents used in our center are either the lymphocyte-depleting agent rabbit antithymocyte globulin (ATG) or the anti-IL2 receptor mAb basiliximab, which is non-depleting.

**Figure 5:**
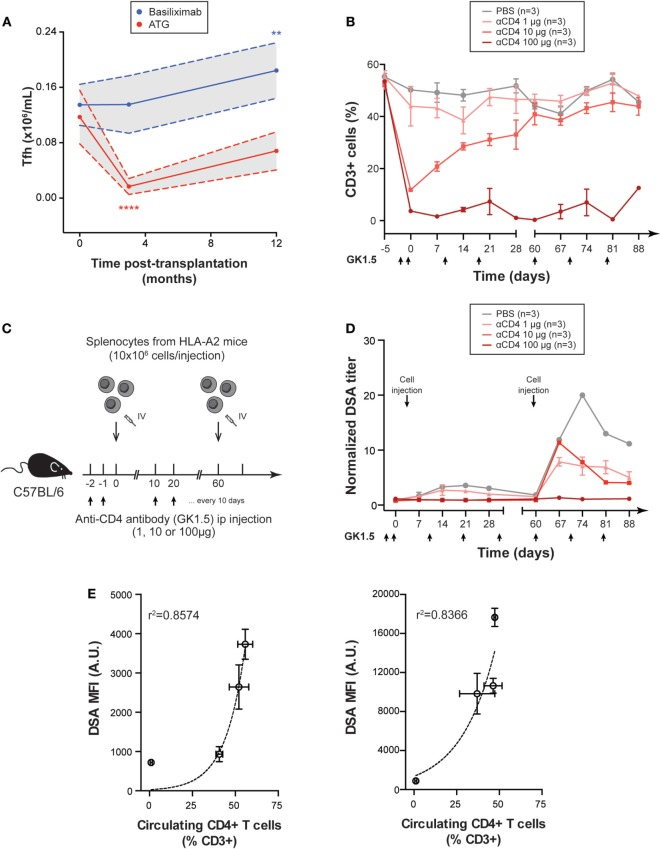
Reduction of CD4+ T cells number dampens donor specific antibody (DSA) responses. **(A)** The number of circulating T follicular helper cells was evaluated in the cohort of 22 renal transplanted patients by flow cytometry and compared according to the nature of immunosuppressive drug used for induction: rabbit anti-thymoglobulin (anti-thymocyte globulin, red line; *n* = 11) or basiliximab (blue line; *n* = 11). Gray areas indicate 95 confidence intervals. Friedman paired test with Dunn’s multiple comparisons. **p < 0.01; ****p < 0.0001. **(B)** Various doses of anti-CD4 mAb GK1.5 were sequentially administered IP to wild-type C57BL/6 mice to obtain various level of CD4+ T cell depletion (*n* = 3 mice per group). Black arrows indicate the timing of mAb administrations. Evolution of the % of CD3+ cells in peripheral blood mononuclear cell (PBMC) (mean ± SD) over time is shown in the control (PBS) and the three experimental groups. **(C)** Graphical representation of the experimental setting used to evaluate the impact of the reduction of circulating CD4+ T cells number on naive and memory DSA responses. C57BL/6 mice were depleted with various dose of anti-CD4 mAb GK1.5 and sensitized at day 0 (naive response) and day 60 (memory response) by IV injection of 10 × 10^6^ PBMC from A2 transgenic donors. **(D)** Evolution of normalized DSA titer (mean ± SD) in the circulation of the control (PBS) and the three experimental groups with various level of CD4 + T cell depletion is shown. **(E)** The relation between the number of circulating CD4 + T cells and DSA titer is shown for naive (left panel) and memory (right panel) responses (mean ± SD). Exponential regression models are plotted (dashed line).

Induction with ATG induced a 90% drop in the number of cTfh at 3 months (Figure 5A). Beyond this time point, the number of cTfh started to increase slowly and was no longer significantly different from baseline at 1 year posttransplantation (Figure 5A). As expected, induction with the non-depleting agent, basiliximab was not followed by any reduction in cTfh number. At the contrary, cTfh number was significantly increased (+36%) at 1 year posttransplantation in patients that received basiliximab (Figure 5A).

Our previous results indicate that while maintenance immunosuppression does not completely block Tfh functions, a prolonged depletion of cTfh can be achieved following induction with a depleting agent. We, therefore, analyzed the impact of such prolonged depletion in CD4+ T cells on naive and memory experimental DSA responses.

GK1.5 (20) is a rat IgG2b anti-murine CD4 mAb commonly used to induce CD4+ T cell depletion in mice (21). Using repeated IV administrations of different doses of GK1.5, we were able to obtain various levels of CD4+ T cell depletion (Figure 5B). Due to (i) the large number of animals to be analyzed and (ii) the complexity of microsurgical models of transplantation (sequential transplantation of two hearts in the cervical area of the same mice is unfeasible, making analysis of memory response impossible), we used a simplified model of allosensitisation (presented in Figure S1 in Supplementary Material). DSA responses of C57BL/6 WT mice were induced by IV injection of purified splenocytes from HLA-A2 transgenic mice (Figure 5C). CD4+ T cell depletion did not change the kinetic of DSA generation but resulted in significant reduction of DSA titers both in naive and memory responses (Figure 5D). Importantly, DSA titers strongly correlated with the number of remaining CD4+ T cells of recipient mice in an exponential regression model (*r*^2^ for naive and memory responses, respectively, 0.86 and 0.84, Figure 5E).

### Long-term Effect of ATG Induction on Tfh Functionality

**Figure 6:**
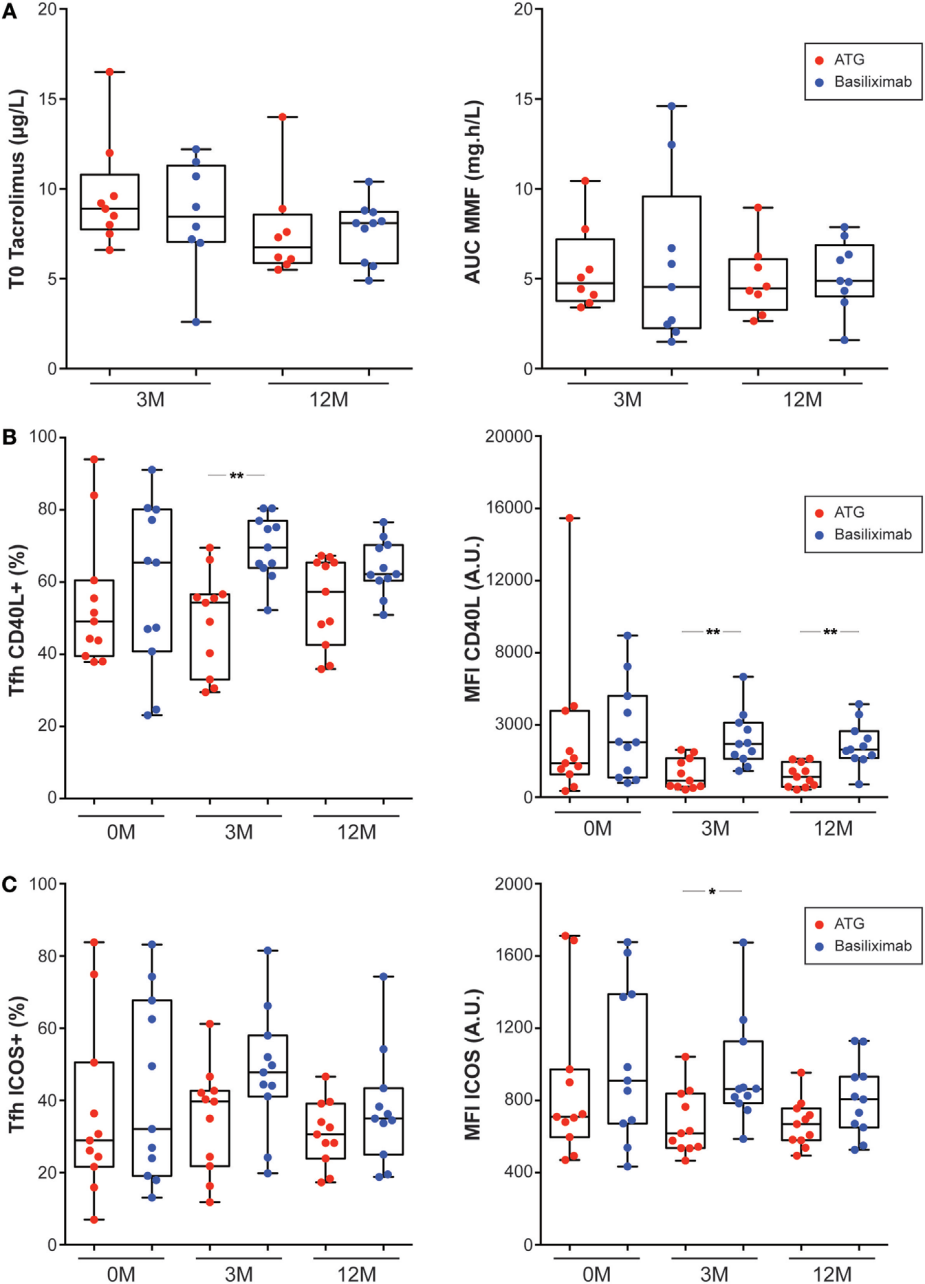
Antithymocyte globulin (ATG) induction has long-term impact on T follicular helper cells (Tfh) functionality. **(A)** Tacrolimus trough levels (T0; left panel) and areas under the curve (AUC) of mycophenolate mofetil (right panel) measured 3 months (3 M) and 12 months (12 M) after transplantation were compared for renal recipients that received induction by ATG (red symbols) or basiliximab (blue symbols). **(B,C)** Flow cytometry was used to analyze the expression of helper molecules CD40L **(B)** or ICOS **(C)** on Tfh surface after 24 h culture in patient’s own plasma and stimulation with CD3/CD28 microbeads. Left panel: percentage of activated Tfh that express the helper molecule. Right panel: level of expression of the helper molecule (mean fluorescence intensity, MFI). Analyses were performed just before transplantation [end stage renal disease (ESRD)/no immunosuppressive drugs, 0 M] and 3 months (3 M) and 12 months (12 M) after renal transplantation (no ESRD/on immunosuppression). Renal recipients that received induction by ATG (red symbols) were compared to patients that received induction by basiliximab (blue symbols). Kruskal–Wallis with Dunn’s multiple comparisons. *p < 0.05; **p < 0.01.

Recovery of peripheral T-cell counts, including cTfh occurred gradually after cessation of ATG treatment (Figure 5A). However, beyond the quantitative impact of ATG, several studies have documented a long-term qualitative influence of the drug on T cell compartment (40). To determine if ATG induction could have long-term effects on Tfh function, we compared the profile of cTfh of renal recipients that received ATG or basiliximab as induction. The two groups did not differ regarding their clinical characteristics (Table 1) or maintenance regimen (Table 1; Figure 6A). Although cTfh functions of the two groups were similar before transplantation (Figures 6B,C), cTfh of renal recipients that received ATG exhibited a significantly reduced activation-induced upregulation of CD40L (Figure 6B) and a similar trend was observed for ICOS (Figure 6C).

These results suggest that ATG induction could reduce the incidence of *de novo* DSA after renal transplantation. We were, however, unable to test this hypothesis in this study because only three patients of the 22 enrolled, have developed *de novo* DSA over the follow-up period (2 in the group ATG induction, 1 in the group basiliximab induction).

## Discussion

In this translational study, we confirmed that CD4+ T cells are essential for DSA generation in a murine model of MHC-disparate skin graft rejection (27). In contrast with grafting procedure (in which allosensitisation of recipient’s immune system takes place in the nearby draining lymph node), organ transplantation leads to the accumulation of donor-derived alloantigens in the spleen of recipients. While this condition was previously reported to abolish the dependence on T cell help for the generation of antibody against viral protein antigens (13, 30), neither naive nor sensitized mice developed DSA after heart transplantation in the absence of CD4+ T cells. These data along with other recent experimental studies (41, 42) demonstrate that CD4+ T cell help is mandatory for both naive and memory DSA responses after transplantation.

It implies that transplanted patients that develop DSA must have maintained a certain level of CD4+ T cell functionality despite immunosuppressive drugs. To test this theory, we analyzed the impact of therapeutic immunosuppression on circulating CD4+ T cells in a cohort of 22 renal transplanted patients. In contrast with CD8+ T cells, exposure to the cocktail of drugs used for maintenance immunosuppression did not change significantly the ability of CD4+ T cells to upregulate the activation marker CD25 upon CD3/CD28 stimulation. Further analyses, focused on Tfh, the subset specialized for providing help to B cells (33, 34), confirmed that maintenance immunosuppression only partially reduced CD40L and ICOS expression on activated Tfh surface and had no effect on their secretion of IL-21. These findings provide molecular explanations as to why cTfh isolated from renal transplanted patients retain the capacity to induce B cell differentiation into immunoglobulin-producing plasmablasts *in vitro*, as recently reported by an independent group (43).

Altogether our data suggest that, beyond non-adherence to immunosuppressive drugs, which has been identified in ~50% of patients that develop AMR (5, 8), the inability of maintenance immunosuppression to adequately block Tfh helper function is also a major cause for DSA generation posttransplantation.

While maintenance immunosuppressive drugs cocktail does not block Tfh function, our data indicate that it is possible to achieve prolonged Tfh depletion in patients if ATG is used as induction therapy. Interestingly, murine experimental model revealed an exponential positive correlation between the number of CD4+ T cells and DSA titers generated both in primary and memory alloimmune responses. Futhermore, beyond its quantitative impact, ATG might also have a beneficial long-term influence on Tfh functionality after reconstitution. Indeed, cTfh of renal transplanted patients induced with ATG exhibited significantly reduced activation-induced upregulation of CD40L and ICOS as compared with cTfh of patients induced with basiliximab. These experimental findings are consistent with the clinical observation by Brokhof et al., which, as compared to basiliximab, induction with ATG is associated with a reduction in the occurrence of *de novo* DSA and AMR in a cohort of 114 renal transplant recipients (3, 44).

We conclude that CD4+ T cell help is mandatory for both naive and memory DSA responses after transplantation and is not adequately blocked by current maintenance immunosuppressive drugs. Why these drugs, which are so effective in blocking CD8+ T cell-mediated cellular rejection, do not show similar efficiency on CD4+ T cell helper function is an important issue that future molecular studies will have to elucidate. Specific pathways involved in helper functions of Tfh indeed represent attractive targets for improving the prevention of DSA generation, and thereby to prolong allograft survival. In this regard, positive preliminary results reported with strategies aiming at blocking costimulation (45) or IL21 pathway (43) represent promising attempts. Waiting for these innovative treatments to be translated into the clinical field, it is important to keep in mind that ATG induction could be effective to limit DSA response after transplantation through both quantitative and qualitative impacts on Tfh subset.

## Ethics Statement

This study was carried out in accordance with French legislation on biomedical research. All subjects gave written informed consent in accordance with the Declaration of Helsinki. The protocol and the biocollection were authorized by the Ministry of Research and the Rhône-Alpes Regional Health Agency (#AC-2011-1375 and #AC-2016-2706). This study was carried out in accordance with the French legislation on live animal experimentation. The protocol was approved both by local “CECCAPP” (http://www.sfr-biosciences.fr/ethique/experimentation-animale/ceccapp) and national AFiS ethical committees (#02870.01).

## Author Contributions

Conception and design of the experiments: C-CC, AK, OT. Acquisition, analysis, and interpretation of data: C-CC, AK, CS, SD, GR, TB, MT, DC, OT. Drafting the MS: C-CC, MO, EM, TD, and OT.

## Conflict of Interest Statement

The authors declare that the research was conducted in the absence of any commercial or financial relationships that could be construed as a potential conflict of interest.

## Supplementary Material

Figure S1 Intravenous allogeneic cell injection model. **(A)** Upper panel: cell suspension was prepared from the spleen of a GFP transgenic mice and 10 × 10^6^ GFP + splenocytes were injected intravenously (i.v. red) or subcutaneously (s.c. blue) to wild-type C57BL/6 mice. Lower panel: 2 days post-procedure the percentage GFP+ cells was enumerated by flow cytometry in the secondary lymphoid organs of recipients (each symbol represents a mice; mean ± SD is indicated). **(B)** Upper panel: cell suspension was prepared from the spleen of an HLA A2 mice and 10 × 10^6^ splenocytes were injected intravenously (red, *n* = 3) or subcutaneously (blue, *n* = 3) to wild-type C57BL/6 mice. Lower panel: evolution of normalized donor specific antibody (DSA) titer (mean ± SD) in the circulation of recipients is shown. **(C)** Graphical representation of the experimental setting used to evaluate the importance of CD4 + T cell help in memory donor-specific antibody response. **(D)** Evolution of normalized DSA titer (mean ± SD) was monitored in the circulation of three groups of recipients sensitized by IV injection of A2 splenocytes: RAG2 KO transferred with anti-A2 memory B and CD4+ T cells (red line; *n* = 3), RAG2 KO transferred with anti-A2 memory B cells alone (red dashed line; *n* = 3) and untransferred RAG2 KO (gray line; *n* = 3).
