# Supplementary figures and images for: CD4+ T Cell Help Is Mandatory for Naive and Memory Donor-Specific Antibody Responses: Impact of Therapeutic Immunosuppression
Source: Front Immunol. 2018 Feb 19;9:275. doi: 10.3389/fimmu.2018.00275 (PMC5825980; doi:10.3389/fimmu.2018.00275)

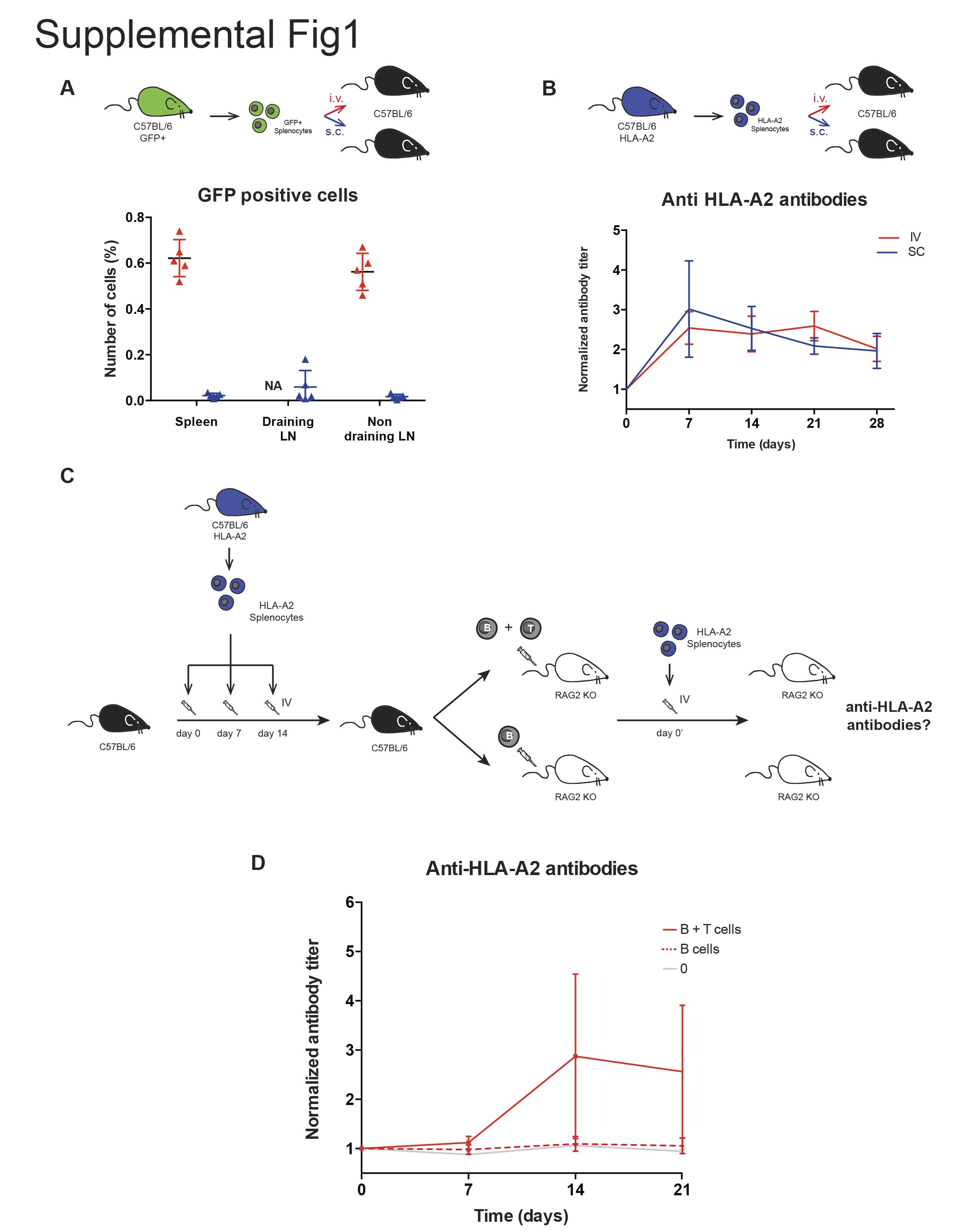

Supplement: Figure S1 — Intravenous allogeneic cell injection model. (A) Upper panel: cell suspension was prepared from the spleen of a GFP transgenic mice and 10 × 106 GFP + splenocytes were injected intravenously (i.v. red) or subcutaneously (s.c. blue) to wild-type C57BL/6 mice. Lower panel: 2 days post-procedure the percentage GFP+ cells was enumerated by flow cytometry in the secondary lymphoid organs of recipients (each symbol represents a mice; mean ± SD is indicated). (B) Upper panel: cell suspension was prepared from the spleen of an HLA A2 mice and 10 × 106 splenocytes were injected intravenously (red, n = 3) or subcutaneously (blue, n = 3) to wild-type C57BL/6 mice. Lower panel: evolution of normalized donor specific antibody (DSA) titer (mean ± SD) in the circulation of recipients is shown. (C) Graphical representation of the experimental setting used to evaluate the importance of CD4 + T cell help in memory donor-specific antibody response. (D) Evolution of normalized DSA titer (mean ± SD) was monitored in the circulation of three groups of recipients sensitized by IV injection of A2 splenocytes: RAG2 KO transferred with anti-A2 memory B and CD4+ T cells (red line; n = 3), RAG2 KO transferred with anti-A2 memory B cells alone (red dashed line; n = 3) and untransferred RAG2 KO (gray line; n = 3). [file image_1.tif]
